# CyberKnife: A new paradigm in radiotherapy

**DOI:** 10.4103/0971-6203.62194

**Published:** 2010

**Authors:** Gopalakrishna Kurup

**Affiliations:** Apollo Speciality Hospital, 320, Mount Road, Nandanam, Chennai - 600 035, India. E-mail: pgg_kurup@yahoo.com

CyberKnife is a stereotactic radiosurgery (SRS) system. SRS is a combination of principles of stereotaxy, or three-dimensional target localization, and radiation beams from multiple directions cross-firing the tumor precisely. Due to the high degree of precision, it is possible to deliver very high dose of radiation to the target with minimal damage to the normal tissues and structures surrounding the tumor. The ideal aim is to ablate the tumor with high radiation dose noninvasively. It has been proved to be an effective alternative to surgery for small tumors and selected medical conditions.

The concept of radiosurgery was developed and put into practice by Dr. Lars Leksell, a Swedish neurosurgeon, in early 1950s.[1] A new device was developed exclusively for radiosurgery with the help of approximately 201 numbers of pencil-type Co-60 sources focused on the region of abnormal brain tissues. These sources were spherically distributed in a helmet around the skull, which is fitted onto the patient's head, and the system is called Gamma Knife.[2] With this system, a relatively spherical dose focused around the target volume, with minimal dose to the surrounding normal cells, could be delivered. The limitation of the Gamma Knife is mainly in its usability outside the head, even though the new generation of Gamma Knife can treat tumors up to C2 vertebra level.

The ‘linear accelerator’–based radiosurgery was pioneered in the mid-1980s. These systems differ from the original Gamma Knife system in many ways. 6-MV x-rays produced from linear accelerator are used in these systems, instead of gamma rays from Co-60 sources. All these systems were used in conjunction with a stereotactic frame fixed onto the skull of the patient. Three-dimensional coordinates of the tumor were precisely determined by the stereotactic frame, and the radiation was delivered accurately to the target volume. Small circular collimators fitted at the end of the treatment head as tresiary collimators were used for narrowing the beam of x-rays from a conventional linear accelerator. Subsequently mini-multileaf collimator (MMLC) replacing the circular collimators enhanced the stereotactic procedures by way of treating irregular lesions in the brain. All these systems require a stereotactic frame to be fixed onto the patient's head, right from imaging to treatment, and hence are termed as invasive or minimally invasive procedures. These methods of conformal dose delivery to the target reduce radiation dose to the surrounding normal tissues substantially.[3,4]

The CyberKnife concept, invented by Dr. John Adler, a neurosurgeon in Stanford, USA, came into practice by 1990s.[5,6] A lightweight linear accelerator fitted onto an industrial robot makes treatment possible precisely in a desired way. The robotic arm has 6 degrees of freedom of movement; unlike the conventional linear accelerator, which has only rotational movement in one plane. CyberKnife treatments are non-isocentric, where beams can be directed from any desired angle. This system does not require a rigid frame to be fixed onto the skull of the patient for stereotactic setup and verification. Initially the CyberKnife was put into use for treatment of only intracranial lesions, like other stereotactic systems.[4] Subsequent developments made it possible to extend the facility to extracranial lesions also, thereby making it a whole-body stereotactic radiotherapy system.

One should understand how the CyberKnife is different from other stereotactic radiosurgery systems. There are a few unique features in CyberKnife to track the tumor precisely and deliver radiation accurately as desired.[7] Unlike other linac-based systems, which have accuracy in millimeters, the CyberKnife has sub-millimeter accuracy in tracking tumor position. If sub-millimeter accuracy is not achieved, it gives warning and stops treatment. The orthogonal x-ray images are taken before each beam and verified for accuracy.

There are 5 different tumor-tracking facilities in CyberKnife treatment. They are 6D skull, fiducial, X sight spine, X sight lung with synchrony, and fiducial with synchrony. These tracking methods are used in different types of sites and with various natures of the organ to be treated. 6D skull tracking is used only for intracranial lesions. Fiducial tracking can be used for any other site. The synchrony tracking feature is used for tracking any moving target in a phased manner with breathing cycles.[8,9] Unlike other systems, where treatment is given on a certain fixed phase of breathing (gated therapy), with synchrony method, the robot can move in synchrony with chest movement during breathing and deliver radiation without interruption as if the tumor is locked to the beam.

Now, the CyberKnife, with its advanced technologies and complexities, gives maximum flexibility in its use to treat any lesion in any part of the body noninvasively. The CyberKnife also has limitations. One of the major limitations is the prolonged treatment time: approximately 30 to 60 minutes. This does not alter the accuracy, because of repeated verifications before each beam delivery. Large volumes are not suitable with CyberKnife as the principle of delivery is ‘dose painting’ from one edge of the tumor to the other. This is most suitable for recurrent and residual tumors after prior radiotherapy treatments. Other than brain lesions, small tumors in lung, liver and spine can be effectively treated using CyberKnife.

A high degree of understanding and training is essential and is emphasized before the system is put to use in clinical practice by any center. The team consisting of radiation oncologists, medical physicists and technologists should understand the principle and technological features of the CyberKnife. Clinicians should understand the tumor biology of short-course treatment with high dose/ fraction; physicists should know the accuracy and dose-delivery principles; and technologists should know the principles, the accuracy desired and achievable and consequences of errors corrected/ liberalized and their impact on treatment delivery.
